# Ethical issues in publishing in predatory journals

**DOI:** 10.11613/BM.2017.031201

**Published:** 2017-10-15

**Authors:** Lorraine E. Ferris, Margaret A Winker

**Affiliations:** 1 Dalla Lana School of Public Health, University of Toronto, Toronto, Canada; 2 Secretary, World Association of Medical Editors (WAME)

This is a correction for *Biochemia Medica* 2017;27(2):279-84. DOI: https://doi.org/10.11613/BM.2017.030. 

This Erratum corrects the section Potential conflict of interest, which was originally published. The publisher apologizes for this error.

